# STRchive: a dynamic resource detailing population-level and locus-specific insights at tandem repeat disease loci

**DOI:** 10.1186/s13073-025-01454-4

**Published:** 2025-03-26

**Authors:** Laurel Hiatt, Ben Weisburd, Egor Dolzhenko, Vincent Rubinetti, Akshay K. Avvaru, Grace E. VanNoy, Nehir Edibe Kurtas, Heidi L. Rehm, Aaron R. Quinlan, Harriet Dashnow

**Affiliations:** 1 Department of Human Genetics, University of Utah, Salt Lake City, UT USA; 2 Program in Medical and Population Genetics, Broad Institute of MIT and Harvard, Cambridge, MA USA; 3 Pacific Biosciences of California, Menlo Park, CA USA; 4 Department of Biomedical Informatics, University of Colorado Anschutz Medical Campus, Aurora, CO USA; 5 Ambry Genetics, Aliso Viejo, CA USA; 6 Center for Genomic Medicine, Massachusetts General Hospital, Boston, MA USA

## Abstract

Approximately 8% of the human genome consists of repetitive elements called tandem repeats (TRs): short tandem repeats (STRs) of 1–6 bp motifs and variable number tandem repeats (VNTRs) of 7 + bp motifs. TR variants contribute to several dozen monogenic diseases but remain understudied and enigmatic. It remains comparatively challenging to interpret the clinical significance of TR variants, particularly relative to single nucleotide variants. We present STRchive (http://strchive.org/), a dynamic resource consolidating information on TR disease loci from the research literature, up-to-date clinical resources, and large-scale genomic databases, streamlining TR variant interpretation at disease-associated loci.

**Supplementary Information:**

The online version contains supplementary material available at 10.1186/s13073-025-01454-4.

## Background

Tandem repeats (TRs) include short tandem repeats (STRs, 1–6 base pair motifs) and variable number tandem repeats (VNTRs, motifs of 7 + base pairs). These two highly mutable classes combined comprise approximately 8% of the human genome and cause numerous human diseases [1–6]. STRs alone contribute to dozens of polygenic (e.g., coronary heart disease) and monogenic (e.g., Huntington’s disease) diseases, with more than 60 Mendelian diseases caused by STR expansions [7–9]. These STR conditions are estimated to collectively affect 1 in 3000 people, with most disease burden presumed to be in undiagnosed individuals [10]. 

This presumption reflects the unique challenges of TR variant detection and interpretation. TRs remain understudied and “enigmatic” [1], particularly when compared to single nucleotide variants (SNVs). Long-standing difficulties analyzing repetitive sequences stem from mappability issues inherent to these low-complexity genomic regions: it is challenging to confidently assign repetitive sequences to the reference genome without distinguishing higher complexity sequences [11]. TRs, thus, have been historically overlooked due to technical challenges in genotyping, even after the advent of next-generation sequencing [12, 13]. Short-read sequencing remains problematic because TRs often approach or exceed the length of the read [14, 15]. While long-read sequencing offers technical improvements through expanded read length, obstacles to genotyping include stutter “noise” from polymerase during sequencing, or a distribution of allele sizes around the original allele, and low coverage leading to limited read support [16]. Consequently, TRs are often excluded from routine genetic studies, or only well-established loci are considered [16, 17]. As TRs have long been proposed to address some of the “missing heritability” in genetic disease [18], their continued absence in research and clinical efforts is a major shortcoming [19, 20]. In fact, the recently discovered STR loci in *RFC1* and *FGF14* have explained a high proportion of previously undiagnosed clinical cases with late-onset ataxia [9]. As stated by Treangen and Salzberg [21], “simply ignoring repeats is not an option.”

However, even when TRs are included in genetic assays, interpreting variants remains difficult. Established filtering strategies (e.g., leveraging inheritance patterns, sequencing depth, and presumed functional impact [14, 22]) can empower some interpretation, but the added complexity of TRs challenges many filtering norms. While many of these variants exist within the coding space of the genome, filtering TR loci to coding regions risks missing TRs with potential functional impact in non-coding regions. Population frequency metrics based on hundreds of thousands of individuals in resources such as gnomAD [23] and TOPMed [24] enable the identification of rare SNVs, which are more likely to be associated with disease [22]. However, normal repeat ranges for TRs have historically been inferred by family studies or control cohorts several times smaller than those used in SNV analyses [25], although larger cohorts such as TR-Atlas are becoming available [26]. Additionally, TRs are exceptionally polymorphic, with 10–10,000-fold higher mutation rates than non-repetitive loci [5]. This extensive mutability can further exacerbate ancestry-specific allelic distributions [17, 27, 28], and large-scale allele frequency distributions are typically unavailable outside well-studied disease loci [25]. Furthermore, most loci are described in European cohorts or small families during disease discovery without capturing the full extent of allellic diversity [29]. Intermediate alleles, or premutations, may correspond to mild, preclinical, or variable phenotypes, such with Fragile X syndrome (FXS) versus late-onset Fragile X-associated tremor/ataxia syndrome (FXTAS) [11, 30]. However, many loci have intermediate allele size ranges for which pathogenicity is ambiguous or unknown due to a paucity of observations. Consequently, the threshold at which TR pathogenicity occurs is frequently unclear and subject to ongoing investigation [11].

These genetic, phenotypic, and diagnostic complexities necessitate the cataloging of TR locus features for diagnostic and research purposes, and efforts have been made as the field develops [14]. A subset of TR diseases are documented in the Clinical Genome Resource [31] and associated variant database ClinVar [32], particularly diseases localized to coding regions. However, the extent of TR-specific documentation is inconsistent and report-dependent, with diagnostic criteria generally unavailable in these resources. GeneReviews [33] offers clinically relevant peer-reviewed information on thousands of genetic conditions—including many TR diseases—but there is a delay from discovery to database inclusion that can last years, and reports differ substantially in detail by disease. Online Mendelian Inheritance in Man (OMIM) [34] has a broadly consistent level of detail for each phenotype-gene relationship; however, its records encompass all variant types rather than providing TR-specific information, and its comprehensive reports can be difficult to parse into discrete, actionable details. None of these tools centralize TR disease loci into a single navigable repository, which is a major strength of the STRipy STRs database [35] and the Genome Aggregation Database (gnomAD) table of TR disease loci [36]. These resources (as of November 25, 2024) include 65 and 60 loci, respectively, with documentation for reference region, canonical repeat motif, and—for most loci—normal versus pathogenic allele ranges. Additionally, both databases have population-level allele distributions stratified by ancestry (2.5 k individuals and five ancestry groups in STRipy; 18.5 k and ten groups in gnomAD). gnomAD also provides the additional granularity of sex, genotyped motif, and, in some cases, sample age. Still, neither STRipy nor gnomAD capture the full information necessary for TR variant interpretation, such as the age of symptom onset, estimated disease prevalence, and theorized pathogenic mechanisms.

We present STRchive (S-T-archive, http://strchive.org/), a dynamic resource that consolidates information on TR disease loci in humans from current literature, up-to-date research findings, and large-scale genomic databases. We combine automated pipelines for literature management with expert curation to ensure STRchive currency and accuracy. STRchive is a comprehensive and version-controlled database that can empower diagnostic efforts and TR research initiatives [17, 19]. Crucially, we interpret the allelic distributions and genotype frequencies in ~ 18.5 k TR disease-unaffected individuals from gnomAD v3.1.3 in the wider context of disease prevalence, clinical phenotype, and diagnostic factors, as distilled within STRchive.

## Construction and content

### STRchive curation and resource management.

STRchive 2.0.0 contains aggregate information on 73 disease-associated loci, including 69 STR and four VNTR disease loci. These are drawn from the literature—including primary reports, case studies, and reviews—and major genomic resources such as OMIM [34] and GeneReviews [33] (Construction and content, Additional File 1: Fig. S1). Beyond the 60 loci documented in gnomAD [36], we present disease-associated tandem repeat loci in *ABCD3* [37], *AFF3* [38], *CBL* [39], *FGF14* [40], *MUC1* [41], *NAXE* [42], *POLG* [43, 44], *pre-MIR7-2* [45], *RAI1* [46], *TAF1* [47], *THAP11* [48], *ZFHX3* [49], and *ZNF713* [50].

Key citations are included within the database, and comprehensive locus-specific literature is cataloged and available to STRchive users. Disease loci were selected based on multiple instances of evidence across the literature and clinical genetics databases, with the first iteration of loci selection conducted on TR review papers [1, 8] and GeneReviews [33]. These loci were then cross-referenced with the Tandem Repeats Finder track [51] in the UCSC Genome Browser [52] to establish a reference region. STRchive locus definitions are generally comparable to those used by gnomAD [36] with a few exceptions (manuscript script CatalogDifferences.ipynb, Additional File 2: Supplementary Methods). These exceptions were explicitly chosen to improve sensitivity when overlapping output from various methods—for example, allowing an imperfect repeat within the sequence when appropriate. While gnomAD locus definitions are calibrated to optimize ExpansionHunter genotyping accuracy [53], STRchive locus definitions endeavor for greater universality in application and broader allelic capture, which sometimes increases reference width. The long-read genotyper TRGT [15] also functions at higher accuracy with wider locus definitions, as genotyping accuracy is reduced when the flanking sequence contains additional repeat variation. We provide TRGT-compatible genotyping input files within the STRchive database as well as bed files aligned to hg37, hg38, and T2T-chm13 reference genomes.

These initial locus details were then augmented by relevant literature, including publications gleaned from manual curation (such as through Google Scholar and PubCrawler alerts), input from clinical and research collaborators, and presentations at publicized genetics conferences. STRchive is available as a user-friendly website and in a machine-readable JSON format for integration into variant calling and analysis pipelines. Within these 73 loci, preliminary loci discovered more recently are annotated with qualifiers, as are loci with sparse or conflicting evidence. Links to locus-specific pages in resources such as OMIM [34], GeneReviews [33], gnomAD [36], and STRipy [35] are provided where available.

STRchive is hosted on GitHub for community involvement and transparency. A user-friendly interface is available through the website strchive.org, which displays and visualizes disease-, locus-, and allele-specific information. Both the Github JSON file and STRchive website contain explicit citations underlying the data included in STRchive, and several scripts are in place to automate data integration where possible—e.g., populating the reverse complementary motifs for negative stranded loci when reference orientation motifs are added.

Resource construction and ongoing maintenance are depicted in Additional File 1: Fig. S1. We provide query code (get-literature.R in the STRchive GitHub) and up-to-date literature directories for the convenience and benefit of STRchive users. We have distilled pertinent information into a comprehensive JSON file and a website-comprehensive table for easy user access. These catalogs will consistently evolve to capture updated loci and facilitate clinical and research endeavors. A version of our diagnostic workflow has been integrated into the Utah NeoSeq project, a collaboration between the Utah Center for Genetic Discovery and ARUP Laboratories to diagnose Neonatal Intensive Care Unit patients [54], as well as into the Undiagnosed Diseases Network, a project funded by the National Institutes of Health to identify genetic etiologies for long-term undiagnosed conditions [55]. The diagnostic blueprint presented was created to synthesize current workflows and considerations implemented through these two partnerships (Table 1).

**Table 1:** STRchive provides a blueprint to aid variant interpretation in a diagnostic workflow. A version of the blueprint that links current resources relevant to each point is available at https://strchive.org/blueprint

Overview	TR-specific details		
**Evaluating allele(s)**			
Allele size	*Premutations*	*Contraction/expansion*	*Somatic mosaicism*
Compare allele of interest to available thresholds for benign, intermediate, and pathogenic size	Evaluate whether an allele may be classified within the intermediate range as a premutation– this may have implications for patient presentation (mild or atypical phenotype) or for family members	While most TR diseases are caused by expansions, contractions are speculated to lead to disease in specific loci where the reference allele is highly constrained. Consider whether an allele may be a pathogenic contraction versus an expansion	Allelic instability may be tissue-specific; evaluate the sampled tissue and whether an allele may be a pathogenic size in the relevant tissue if the allele approaches a pathogenic threshold
Sequence composition	*Motif classification*	*Interruptions*	
Compare genotyped sequence motifs with reference and known TR sequences	Determine if the genotyped motif is benign, pathogenic, or of unknown consequence	Assess motif sequence purity, as interruptions may increase or decrease penetrance, disease severity, or age of onset	
Genotype quality	*Read visualization*	*Technology*	
Check genotype quality and read support to filter unreliable calls	Review read visualizations for alleles of similar size to assess expected read support and pattern of interruptions	Appraise the molecular and sequencing technologies used to identify the allele and how this may impact reliability of calls. For instance, read length strongly impacts genotyping capabilities	
Allele frequency	*Ancestry-specific*	*Polymorphic distribution*	
Determine allele frequency within a broader population; rare mutations are more likely to underlie rare disease	Populations with different ancestries may have allelic distributions that differ from conventionally established or referenced ranges; review the allele in the context of the relevant population if possible	Given the highly polymorphic aspect of TRs, there are far more alleles likely to be present in a population at most loci than variants such as SNVs. As this may deflate exact allelic frequency, consider whether the allele falls outside of the normal distribution of alleles in addition to its exact frequency	
Inheritance pattern	*Mixed mutation types*		
Assess both alleles (if present) in case of recessive condition	Consider non-TR, potentially compounding variants in the second allele		
**Evaluating phenotype**			
Genotype–phenotype correlation	*Anticipation*	*Reduced penetrance*	*Atypical presentation*
Compare clinical history to symptoms associated with gene (if any). Assess whether patient history matches reported disease age of onset range	TR diseases may demonstrate anticipation, where disease severity increases and age of onset decreases by generation as alleles expand through transmission. Consider family history	Penetrance can vary due to genetic modifiers and allelic attributes (motif, interruptions, etc.). Recall that a pathogenic genotype may not indicate current or future disease	TR disease can present with immensely variable phenotypes, both in terms of severity and specific symptoms. Often, there is an inverse correlation between allele size and age of onset, which can lead to early and late-onset diseases outside of the conventional range
**Evaluating the locus**			
Known disease association	*Predicted pathogenicity*		
Evaluate whether the locus has established association with TR disease by comparing to current catalogs	There are loci that, while not associated with documented disease, have been predicted to be pathogenic through machine learning-based predictions. Additionally, manual comparison to known disease loci can inform the prediction of pathogenicity at novel loci based on known mechanisms of disease (e.g., polyalanine/glutamine tracts.)		
Identify whether the gene has previous gene-disease associations documented for non-TR variant types			
Genomic region	*Proximity to another TR locus*		
The genomic region in which a locus is present is highly informative: whether coding/non-coding, whether it overlaps genetic elements such as promoters/enhancers, and whether nearby variants have known disease relevance	Several TR disease loci are found within the same gene. TR locus proximity may indicate potential pathogenicity, but also may lead to inflated allele estimation. Leverage nearby loci to inform variant interpretation		

The complexity and heterogeneity of TR loci means clinical and biological information may not be available in all cases. We recommend reviewing pertinent literature (cataloged by STRchive) and using best judgment when prioritizing variants

### Automated literature retrieval and STRchive additional curation.

Literature for this manuscript was retrieved on November 25, 2024, by searching for genes and gene synonyms acquired through biomaRt in conjunction with tandem repeat-related search terms through the R library easyPubMed—explanation of query refinement and modification and assessment of earliest PubMed publication are available in Additional File 2: Supplementary Methods.

Queried PMIDs were leveraged in addition to OMIM [34], GeneReviews [33], and Orphanet [56] to establish ranges in age of onset (including documented extremes and the typical range), detected motifs with clinical classification, prevalence estimates as available, and a number of independent observations (Additional File 1: Fig. S1). All data incorporated into STRchive and related analyses were restricted to clinical cases explicitly linked to TR expansion. Pathologies sharing an OMIM entry but not exclusive to TR expansion (such as glutaminase deficiency or Duchenne Muscular Dystrophy) were reviewed to include TR-specific clinical cases. When literature was unavailable through query (for example, case reports published before indexing or restricted by language/terminology retrieval), publications were independently retrieved and assessed through interlibrary loan. Specific citations underpinning disease prevalence estimates and ranges in age of onset are included in related STRchive text fields in the full database. Disease prevalences in STRchive are averaged to a singular value when ranges are presented without a consensus prevalence estimate.

**Fig. 1:**
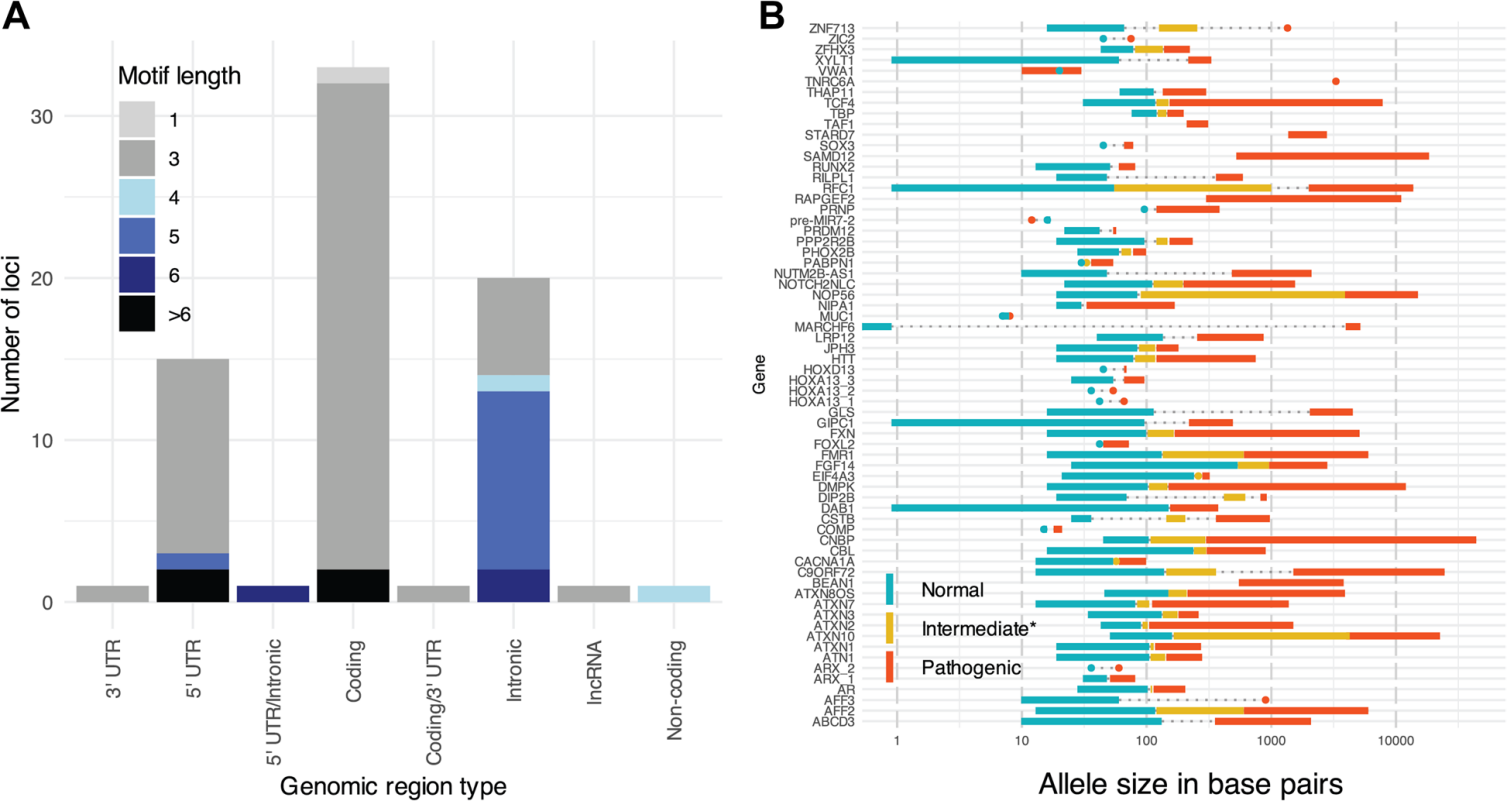
STRchive documents essential information across TR disease loci, from sequence context to locus-specific data.** A** TR locus counts by motif size and genomic context. Additional breakdown of coding loci available in Additional File 1: Fig. S2. Multiple classifications reflect transcript-specific differences. **B** Ranges of literature-established allele sizes in bp (citations available on STRchive). The intermediate size range indicates either a premutation, incomplete penetrance, or an uncertain threshold of pathogenicity; circles indicate a value rather than an interval. Where there are no intermediate values but pathogenic thresholds are greater than the upper limit of the normal thresholds, dashed gray lines have been added. Independent observations are defined as unrelated cases/pedigrees as documented in OMIM, GeneReviews, and research literature; loci with less than two independent observations, or unrelated clinical cases, were removed, as was the *POLG* locus (see the “Construction and content” section)

Disease loci with < 2 independent observations (*DMD*,* ZIC3*,* TNR6CA*,* YEATS2*,* TBX1*,* NAXE*, and *RAI1* as of November 25, 2024) were removed from Figs. 1B and 3, given a lack of literature consensus to support establishing a reference for these loci. Additionally, *POLG* was removed, given the presence of expansions commonly in control/healthy individuals [43].

### Calculating and comparing pathogenic genotypes

We used the genotypes generated in gnomAD by Expansion Hunter [36] at the intersecting STRchive loci to estimate inferred pathogenic genotypes (PGs) based on pathogenic thresholds. For the analyses, the inheritance pattern for *ATXN2*,* FOXL2*, and *PABPN1* was assumed to be autosomal dominant (AD), even though autosomal recessive cases have been seen in certain contexts. All motifs were normalized (nucleotides arranged in alphabetical order) to facilitate motif matching, as genotypes were required to be called with known pathogenic motifs to be considered potentially pathogenic. Loci with the genotyped motif “CNG” were excluded from calculations due to apparent inflation in allele estimates likely due to sequence non-specificity. The specific loci within *AFF2*, *NOTCH2NLC*, *TBP*, *ZNF713*, and *NIPA1* were also removed due to unreliable genotyping calls following manual review. The results underlying these exclusions are discussed in Additional File 2: Supplementary Methods.

The intersected gnomAD/STRchive dataset was subset by inheritance pattern (AD, autosomal recessive, X-linked dominant, and X-linked recessive) and analyzed according to inheritance pattern. Dominant conditions required a single allele to exceed the pathogenic threshold (pathogenic_min) and a matched motif. In contrast, recessive conditions in individuals with two alleles required two inferred pathogenic alleles (exceeding the pathogenic minimum with matched motifs) to have an inferred PG.

The number of PGs was calculated and converted to a percentage with the number of PGs as the numerator and the number of individuals genotyped at the locus as the denominator. A 95% binomial proportion confidence interval for the PG percentage was generated in R by using the number of individuals genotyped for a locus as the number of “trials” and the number of PGs as the number of “successes.”

In our estimates of PGs, we used the allele lower bound estimates for each allele because while there is broad concordance between the genotype and the lower bound estimate (allele estimates were identical in 97.02% of calls for allele 1 and 94.13% of calls for allele 2), Expansion Hunter tends to overestimate alleles when erring and we endeavored to be conservative in our estimates of pathogenicity [13]. Average difference between allele 1 and the lower bound estimate is 0.22 repeat units for all calls and 7.40 (range 1–251, median 6) for the subset where allele 1 is not equal to lower bound estimates. For allele 2, the average distance was 0.42 repeat units for all calls and 7.14 for the subset of non-identical values (range 1–267, median 5). A full analysis script, including merging with STRchive disease prevalence estimates, is available at CalculatingPGsandConfidenceIntervals.R within the manuscript GitHub.

The data of 100 individuals from the Human Pangenome Reference Consortium (HPRC) genotyped in long-read sequencing data by TRGT was provided by coauthor Egor Dolzhenko and used for orthogonal assessment of PGs [15, 57].

Comparison with Ibañez et al. data was performed by comparing their reported PG percentages for intersecting loci to our data set's PG percentage confidence intervals [28]. Evaluation of gnomAD PGs when matching pathogenic thresholds to those used by Ibañez et al. were performed by identical scripts as in our analysis, with the pathogenic minimum substituted for the thresholds used by Ibañez et al. as appropriate.

## Utility and discussion

### STRchive combines automatic and supervised curation for comprehensive cataloging

We developed an automated PubMed search query (detailed in Additional File 2: Supplementary Methods) to systematically update our database with locus-specific literature on a regular basis. This pipeline runs monthly by default, with the flexibility for more frequent updates if needed. A specific query within the pipeline focuses on identifying novel loci, while GitHub discussion pages provide a collaborative space for flagging and assessing new loci and findings that extend beyond the scope of the automated searches. New publications are manually reviewed at least quarterly for established loci and monthly for new loci, and relevant findings are assessed by our team of contributors for inclusion in STRchive. In addition to this automated approach, our curation process is enhanced by ongoing manual literature review and community contributions. This comprehensive approach allows us to catalog detailed information for each disease-associated locus, including genomic location, motif length, and allele size ranges relevant to pathogenicity (Fig. 1).

**Fig. 2:**
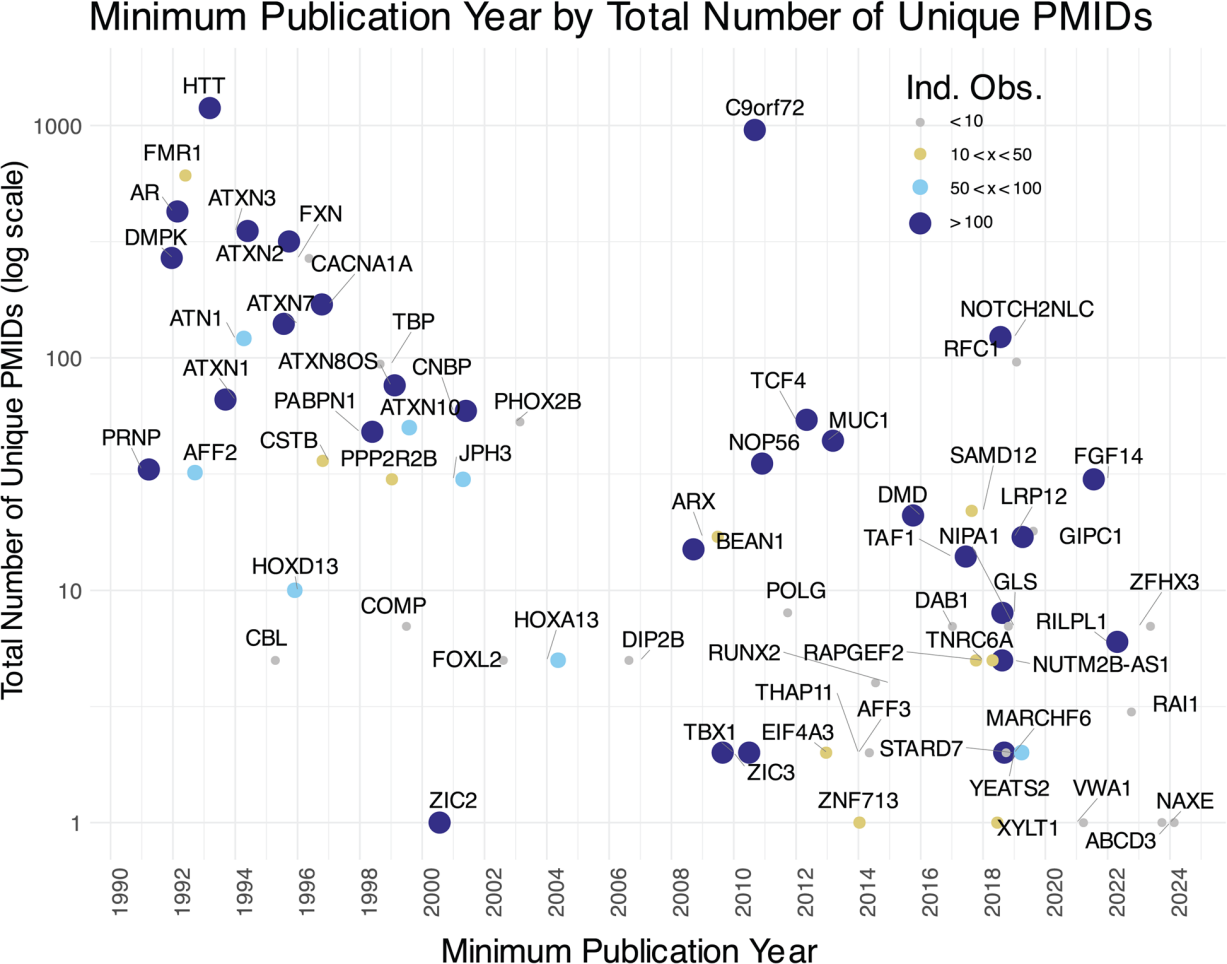
Locus-specific data, from literature catalog to clinical evidence, are captured by automated and manual curation. Total number of PMIDs with available PubMed year of discovery or earliest mention in indexed literature (as of November 25, 2024). Loci are colored and sized by the number of independent observations, defined as unrelated cases/pedigrees as documented in OMIM, GeneReviews, and research literature. Jitter is used to separate data points; years are considered as whole integers

Our automated literature retrieval identifies the earliest PubMed-indexed publication reporting the discovery of an associated monogenic disease at a TR locus. We contrast the number of unique PubMed IDs (PMIDs), including and after the earliest publication, with the number of independent observations (or non-related clinical cases) supporting the disease association, manually curated from the literature (Fig. 2) [1]. These publications were identified by explicit PubMed queries mentioning tandem repeats, human disease, and the locus gene (“Construction and content”). We capture the trend of increased discovery of TR loci as in the past decade as parallels advances in molecular and bioinformatic methods [58].

### STRchive reveals potential for childhood onset for a majority of TR diseases

**Fig. 3:**
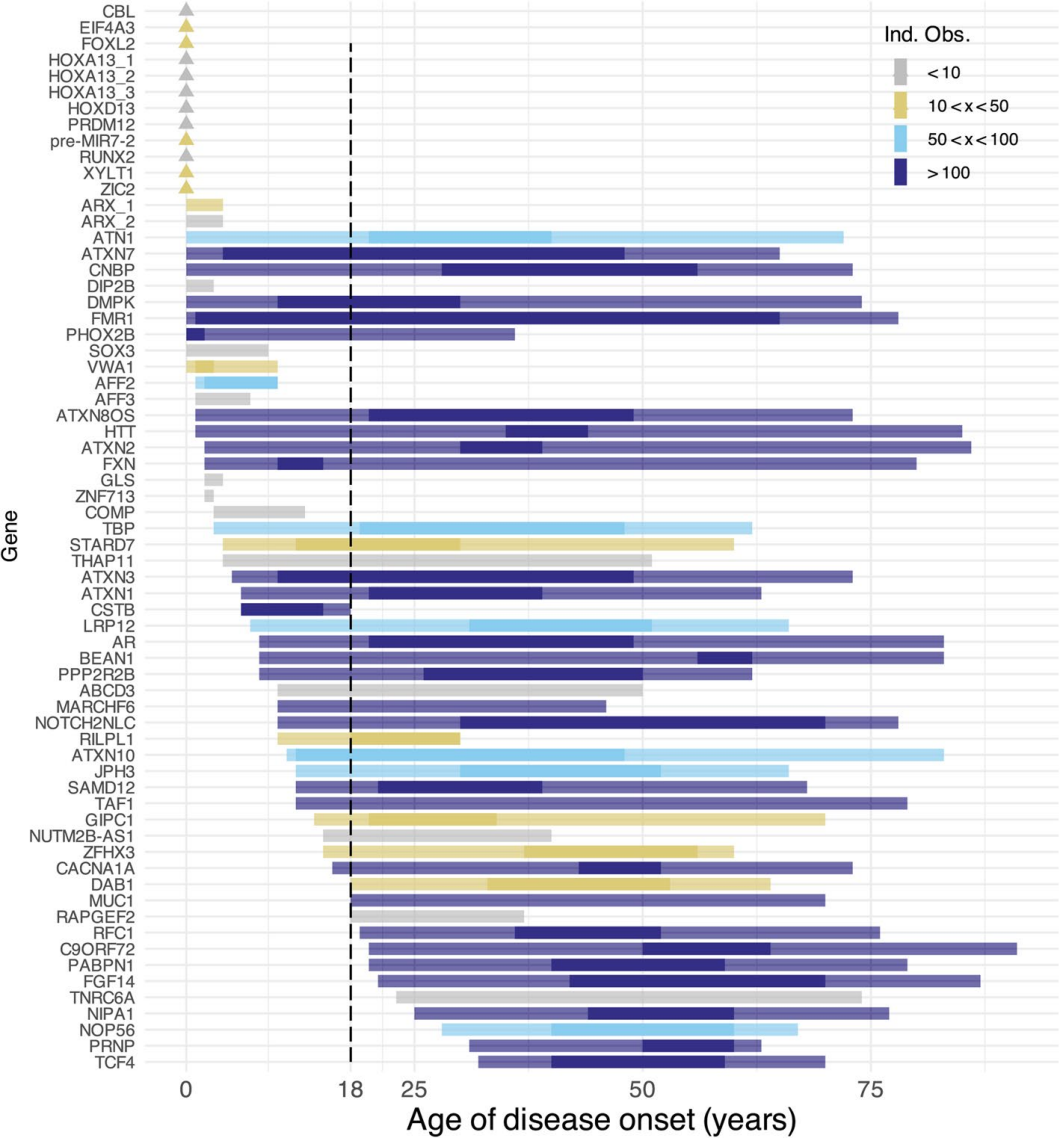
Ages of onset for TR disease, with the majority of loci having possible pediatric onset. Triangles indicate congenital conditions occurring at birth. Loci are colored by the number of independent observations, defined as unrelated cases/pedigrees as documented in OMIM, GeneReviews, and research literature. Lighter bars connect maximum and minimum reported ages, while opaque lines indicate typical intervals for age of onset, where greater than ten independent observations are available. Loci with less than two independent observations were removed, as was *POLG *(see the “Construction and content” section)

While TR diseases are often thought to primarily affect adults due to allele instability over the lifetime [59], 82% (60/73) of documented TR conditions can affect children, with a documented case under the age of 18. Over a third (25/73) can present in the first year of life. To our knowledge, this is the first instance in which sufficient data have been aggregated to challenge the dogma of TR diseases as specific to adults. To determine whether pediatric cases fall within the expected range of disease onset or exist as outliers, we annotate the evidence supporting each locus and assign literature-based typical onset ranges where there are ten or more independent observations (Fig. 3). We observe wide ranges of disease onset for well-documented diseases: the higher the prevalence and penetrance of a disease, the more likely we are to observe age variation due to a greater extent of case documentation.

### Sequence motif complexity is essential to variant interpretation

STRchive annotates motifs detected at each locus by disease-relevant classification: benign, pathogenic, or uncertain significance. For most loci (60/73), the repeat motif in the reference genome (i.e., “reference motif”) is the pathogenic motif, and pathogenicity is conventionally determined by allele size. In the remaining nine loci, the observed motifs differ in pathogenicity, and specific patterns in the expansion may be necessary to cause disease. Some motifs might expand without introducing pathogenicity, while others introduce pathogenicity at lower thresholds [27, 60]. For this reason, we document the locus structure or repeating sequence pertinent to disease for each locus. Although motif consideration is essential in variant interpretation, the biological consequence of motifs is still unknown in the majority of cases. Such subtleties may be overlooked in clinical evaluation and can introduce challenges in PCR-based assays.

### STRchive contextualizes gnomAD population data when assessing TR disease loci

A rational approach to elucidating the details of TR loci (e.g., motif significance or allelic frequency) is by investigating population-level TR data [20]. Empowering such an analysis, gnomAD v3.1.3 recently added allele size estimates at 60 disease-associated TR loci from more than 18,000 individuals using ExpansionHunter; this data is a subset of gnomAD individuals where whole-genome sequencing data was available for TR variant calling. As most TR data are derived from case studies or small cohorts of affected individuals, this database is an invaluable step forward to elucidate locus-specific variation in the general populace. At the same time, each locus presents unique bioinformatic and biological contexts which are necessary to understand when performing variant-, locus-, and phenotype-based analyses.

We leverage comprehensive, locus-specific information from STRchive to assess the gnomAD genotypes, which include motif and allele size estimates. We estimate the fraction of gnomAD populations with pathogenic genotypes (PG) and with carrier status, taking inheritance patterns into account. Only calls where the sequenced motif matched a pathogenic motif are considered pathogenic. We exclude loci genotyped with “CNG”, as these were shown to have inflated allele estimates likely due to the non-specificity of the “N” and proximity to other repetitive sequences (Additional File 2: Supplementary Methods). Given the intrinsic complexity of TR diseases, some simplification was used. Expansion is typically considered necessary for TR pathogenicity. However, loci such as *VWA1* have suggestive evidence of pathogenicity secondary to any deviation from the constrained allele size, whether expansions or contractions [43, 61]. As there is limited evidence for the likelihood of pathogenicity with contractions, the role of modifier alleles, and other such biological circumstances, our analysis was restricted to allelic expansions with pathogenic motifs at non- “CNG” loci with trustworthy genotyping after manual review (“Construction and content”).

**Fig. 4:**
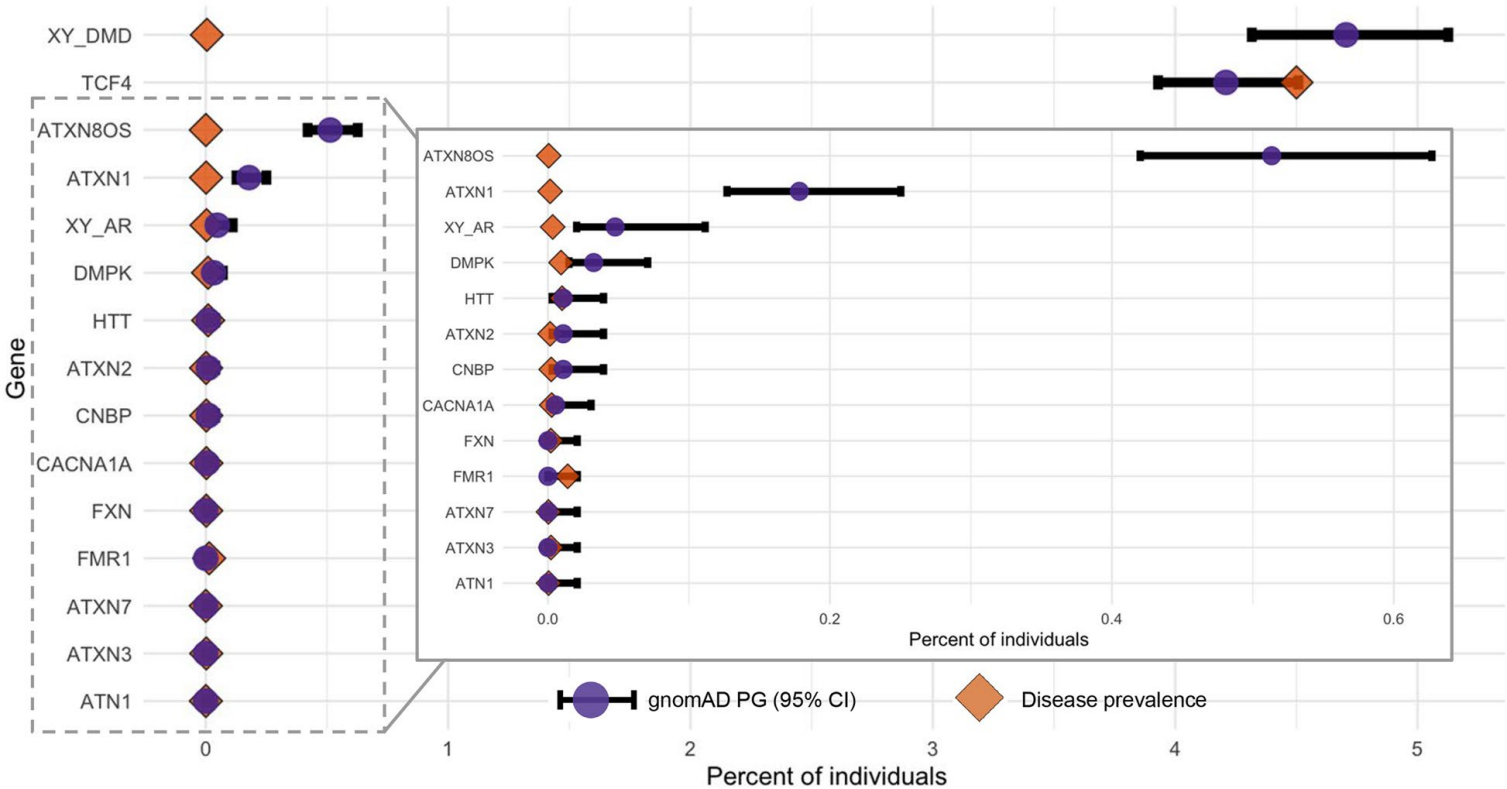
Pathogenic genotypes are found within the presumably unaffected gnomAD cohort, which correspond to and vary from known prevalence dependent on loci. Disease loci where PGs were found have the PG percentage (purple circle) within the gnomAD cohort shown, compared to disease prevalence ascertained by the literature (orange diamond). The PG percentage has a 95% binomial confidence interval calculated and plotted (black bar). Loci where prevalence is unknown are excluded. The inset plot’s *x*-axis is 0.0–0.64

We identify 14 autosomal dominant loci with at least one expanded allele and two X-linked recessive loci with either one expanded allele in males (*DMD, AR*) or two expanded alleles in females (*DMD*) (Fig. 4). Results are contrasted with general disease prevalence in the literature where available (citations available at STRchive.org). We demonstrate cases of robust overlap (such as *TCF4, HTT,* and *ATN1)* as well as cases of separation (*DMD*, *ATXN8OS)* which in turn could imply reduced penetrance, delayed onset, or even questionable pathogenicity. Full calculated results are available in Additional File 3: Table S1.

We now demonstrate the application of STRchive to the diagnostic process by discussing loci within the gnomAD dataset that exhibit unique aspects of TR variant interpretation, noting how these vignettes intersect with our variant interpretation guideline (Table 1). Our guideline and clinical vignettes reflect three overarching themes: evaluating allele(s), evaluating phenotype, and evaluating the locus.

### Evaluating allele(s)

STRchive integrates literature and resources related to *allele frequency*, *inheritance patterns*, and methods of assessing *genotype quality*, in addition to carefully curated information related to allele size and sequence composition.

### Allele size can profoundly inform clinical expectations

TR disease loci are often evaluated in a binary fashion: if the allele exceeds a pathogenic threshold (or two alleles in a recessive condition), it is considered a pathogenic genotype. However, exact allele size is an essential consideration in interpretation, as age of onset and disease severity can be highly variable and correlated with repeat length (Fig. 3). For example, while Huntington’s disease (HD) most typically presents in adults of three to four decades, sufficiently large expansions can cause disease onset in children as young as three years, while smaller pathogenic expansions may lead to disease in elderly individuals with mild symptoms [62]. Years-to-onset trajectory in diseases such as HD may be predicted by allele size, which in turn can be used in risk assessments for children and young adults [63]. In gnomAD, 0.011% (95% confidence interval: 0.003–0.039%, Additional File 3: Table S2) of individuals had at least one *HTT* allele exceeding 39 repeats, which closely matches the prevalence documented in the literature of 0.0106–0.0137% [20, 64]. The presence of PGs in the gnomAD cohort, even with conservative genotype estimates, may reflect the presence of these minimally expanded variants (mean of expanded alleles: 42 repeats) leading to patient ascertainment at a presymptomatic age.

While not ubiquitous, the relationship between allele size and clinical outcome is observed across many TR disease loci [10]. Spinocerebellar ataxia 8 (SCA8) is caused by a CTG expansion and a corresponding, complementary CAG expansion in the overlapping *ATXN8OS* and *ATXN8* genes, respectively [65]. The observed range of pathogenic alleles causing SCA8 is notably wide (71–1300 repeats) and allele length is believed to influence disease penetrance, severity, and progression [65–67]. The SCA8 PG percentage in gnomAD is the second highest frequency for autosomal dominant loci at 0.513% (~ 1 in 200 individuals, 95% CI: 0.420–0.627%), a frequency 1000-fold higher than the estimated literature prevalence for SCA8 [20]. This incongruity reinforces previous research that expanded alleles greatly outnumber disease cases due to reduced penetrance, with intermediate and pathogenic range expansions occurring 1 in 100–1200 chromosomes, depending on the population [67]. As such, comparing the magnitude of an allele against the patient’s age and clinical history is highly informative in the diagnostic process for these and other loci. Referencing the clinical literature cataloged by STRchive can provide points of comparison to set expectations of phenotype.

### Sequence composition is an essential aspect of allele interpretation

**Fig. 5:**
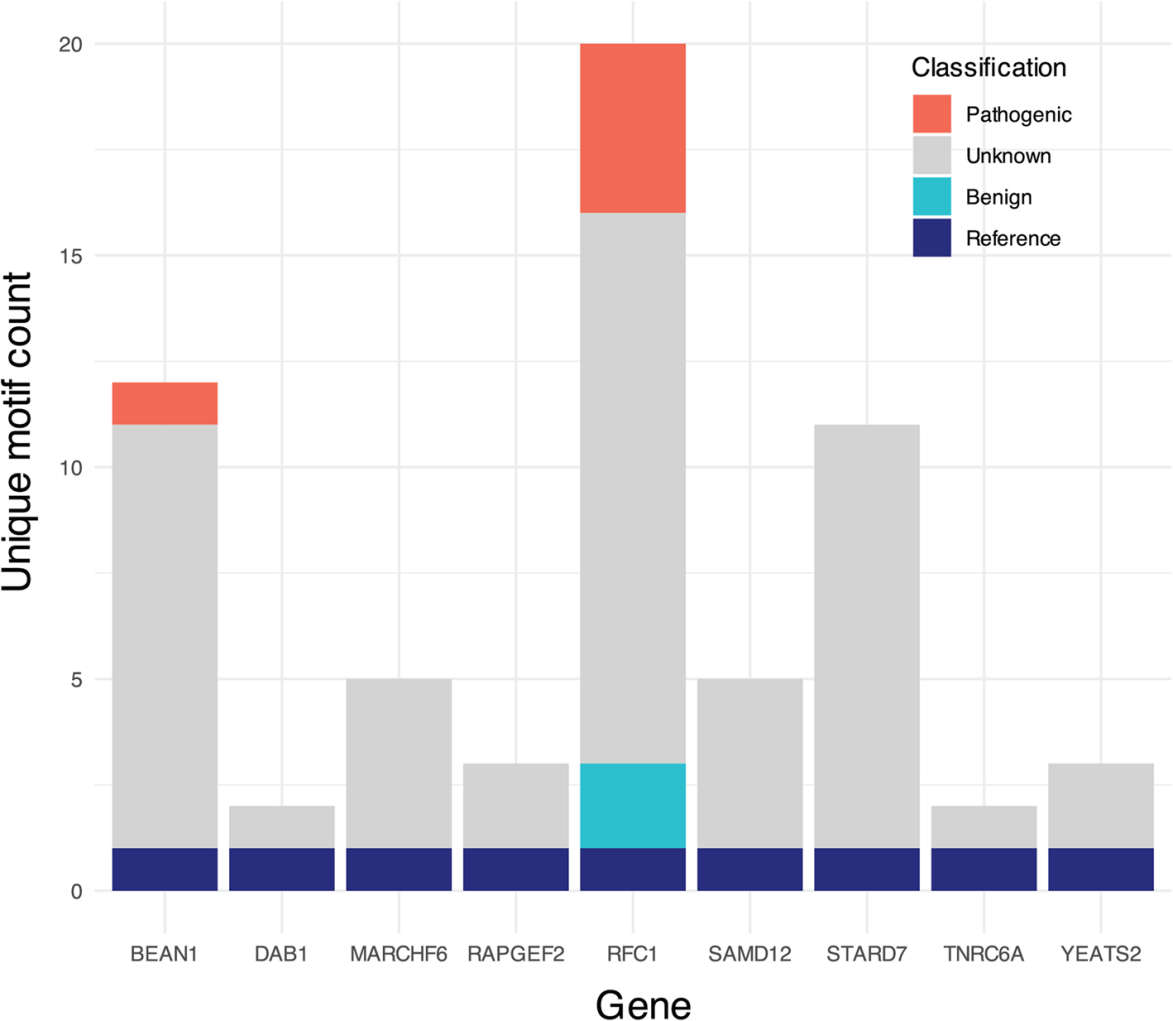
Nine gnomAD loci demonstrate motif heterogeneity, with two possessing pathogenic motifs captured in locus genotypes. Unique gnomAD motif counts where greater than one motif (the reference motif) is present, with STRchive motif classification applied

At least 20 disease loci have shown clinically relevant changes in sequence composition, whether dispersed within a sequence as interruptions, alternating with the canonical motif, or entirely replacing the reference allele with an alternative motif [68]. As such, STRchive documents motifs and records pertinent interruptions as they affect sequence composition, which in turn can impact patient phenotype. Within the gnomAD data set, exactly 15% of loci (9/60) had multiple motifs (2–20) genotyped beyond the reference (Fig. 5). The *RFC1* locus underlying cerebellar ataxia, neuropathy, and vestibular areflexia syndrome (CANVAS) had 20 unique motifs identified, with pathogenic motifs identified with relatively common frequency and shown to have generally longer allele length (Additional File 1: Fig. S3).

The motif diversity at TR loci adds complexity to variant interpretation and is an ongoing area of development, as reflected in our data. Motif consequence is unknown in about 3/4ths of distinct motifs detected at these nine loci (47/63 unique motifs genotyped). Without knowing the association between a motif and a phenotype, or the threshold at which pathogenicity occurs for a specific motif, allelic consequence is challenging to determine. Motif heterogeneity is common even within a smaller cohort: we identify unique motifs from 100 individuals from the Human Pangenome Reference Consortium (HPRC) genotyped in long-read sequencing data by TRGT [15, 57]. Six gnomAD loci with multiple motifs also had multiple motifs in the HPRC data (*BEAN1*,* RAPGEF2*,* RFC1*,* SAMD12*,* STARD7*,* YEATS2*; Additional File 1: Fig. S4*)*. Four additional loci showed motif heterogeneity in the HPRC data (*FGF14*,* XYLT1*, *ZFHX3*,* C9orf72*), with none of the non-reference motifs of these four loci having documented classification in STRchive (Additional File 1: Fig. S5). These findings highlight the importance of ongoing motif documentation within STRchive as information becomes available about motifs’ phenotypic implications.

In addition to motifs, interruptions within a sequence can greatly impact phenotype. *ATXN8OS* interruptions are known to influence disease status and severity in SCA8 [65, 69]. Specifically, interruptions within the CAG tract appear to increase penetrance and protein toxicity [69]. As affected and unaffected individuals can have *ATXN8OS* expansions (as reinforced by our dataset) [67], the SCA8 locus further exemplifies the need to consider sequence composition in variant interpretation. Sequence composition changes may complicate variant interpretation on a bioinformatic level by impacting detection performance and genotyping accuracy [16, 17]. Interruptions may inflate allelic estimate, and an expansion may be missed if the correct motif is not targeted during genotyping [11]. By documenting sequence composition changes, STRchive endeavors to facilitate TR detection in addition to aiding diagnosis.

### Allele frequency within a population can inform expectations of pathogenicity

Although we do not evaluate the exact allelic frequency of TRs within a population given their polymorphic nature, we assess the frequency of PGs in a population presumed to be unaffected by TR disease. While gnomAD presents a larger cohort to assess disease genotype than many of the family studies in TR literature, TR diseases are rare and each specific disease typically affects far fewer than one in 20,000 individuals. Thus, most disease loci with full penetrance would not be expected to have PGs in this cohort of ~ 18.5 K individuals. Of the four disease loci where a PG is feasible by prevalence alone (estimated ≥ 1 in 18,500: *DMPK*, *HTT*, *FMR1*, *TCF4*), all but *DMPK* had a PG confidence interval spanning the documented literature prevalence. This highlights the necessity of considering allele frequency specifically, rather than solely disease prevalence: our *DMPK* findings (0.0324%, 95% CI: 0.0149–0.0707%) are comparable to one study’s frequency of *DMPK* repeat expansions taken from more than 50,000 newborn screenings (0.0476%, 0.0286–0.0667%) [70]. This suggests that *DMPK* expansions are present in the general population even at birth, and may pose as incidental or secondary findings. While genotyping inaccuracy in particularly large alleles could potentially lead to size underestimation, all *DMPK* PGs in the gnomAD cohort are within the “mild” expansion range of the disease which can lead to disease as late as age 70 [71].

The gnomAD *DMPK* data also matches prevalence estimates ascertained within specific populations of elevated prevalence (such as Iceland), which may indicate population specificity which in turn can result in different allele frequencies [71, 72]. Allelic frequencies should be considered in the context of patient ancestry, which may impact the distribution of TR variant sizes. However, prevalence rates and allele frequency estimates are unavailable for many TR disease loci given heterogeneous clinical presentations, variable population ancestries, and technical limitations [28]. Rarer TR diseases likely require a larger population cohort for sufficiently granular resolution establishing allelic frequency as well as more certainty about genotype accuracy to meaningfully compare to prevalence.

Disparities between large cohort PGs and clinically based disease prevalence estimates have been noted previously. In a study leveraging TOPMed and the 100,000 Genomes Project (100kGP) to genotype STR disease loci across ~ 82 k individuals, Ibañez et al. estimated that TR diseases likely affect up to three times more individuals than currently recognized clinically [28]. Of the thirteen loci surveyed by Ibañez et al. also in the gnomAD dataset, twelve had PG estimates concordant with our data—defined as a cohort estimate within or within 0.001% of the gnomAD 95% confidence interval—when using the same pathogenic thresholds (Additional File 1: Fig. S6, Additional File 3: Table S3). Only one locus was discordant: *FXN*, known to have ancestry-specific disease prevalence [73]. To resolve the ambiguities presented by the above discordances and associated research, STRchive will continue to record prevalence estimates and allelic frequencies as derived, which can be used in turn to evaluate the likelihood of a variant’s pathogenicity.

### Evaluating phenotype

STRchive catalogs extensive literature describing clinical cases and assorted genotype-phenotypes. Links to important clinical resources specific to TR diseases are provided within the website, as are comments on factors that may precede atypical clinical presentations. STRchive locus definitions redirect to specific locations within the UCSC Genome Browser, which itself shows overlapping gene phenotypes and can be overlaid with informative tracks [52].

### Informed genotype–phenotype comparisons can lead to candidate inclusion (or exclusion)

Carefully evaluating alleles of interest can inform expectations for phenotype, such as in HD when there is remarkably early- or late-onset of disease based on allele size. Similarly, awareness of changes in sequence composition can explain atypical presentations; for example, “CCG” interruptions within the “CTG” STR expansion in *DMPK* lead to unusual disease traits such as severe axial and proximal weakness, in addition to delayed onset of symptoms [11]. Interruptions such as these may explain some of the presence of *DMPK* PGs in gnomAD exceeding disease prevalence. Trans-genetic elements may modify disease presentation, including non-TR mutations in related genes [74], and epigenetic factors like methylation can influence allele penetrance [8, 29]. There may be phenotypic considerations at loci that extend beyond the allele to the overall disease. “Atypical” presentations may be the norm for loci with tremendous clinical heterogeneity: *NOTCH2NLC “*CGG” expansions are associated with neuronal intranuclear inclusion disease, Alzheimer's disease, essential tremor, Parkinson's disease, amyotrophic lateral sclerosis, and oculopharyngodistal myopathy [75]. Additionally, some loci exhibit anticipation or a worsening of phenotype over generations, increasing the utility of family history. Lastly, reduced penetrance may lead to the complete absence of phenotype even when an expansion is observed. These considerations are complex, and we endeavor to provide robust resources through STRchive to distinguish between non-causative expansions versus expansions leading to atypical phenotypes, as well as flag loci with anticipation and reduced penetrance to inform diagnostic expectations.

Beyond specific symptom matching, evaluating the phenotype of a TR expansion can inform variant prioritization based on expectations of severity. The prevalence estimates documented by STRchive can underscore locus expectations: higher prevalence generally indicates a less deleterious disease. This trend was reflected in the gnomAD data: the highest percentage of PGs in an autosomal dominant condition and the second highest overall frequency in our dataset was 4.21% in *TCF4*, an STR locus causing Fuchs endothelial corneal dystrophy 3 (FECD3) [76]. FECD3 is estimated to affect approximately 4% of the population older than 40 years, with a decades-long disease progression leading to reduced endothelial function and vision impairment. In contrast to many other TR diseases, corneal dystrophy is not expected to reduce lifespan or reproductive success. In fact, FECD3 was originally overlooked as a pathogenic expansion because neurodegeneration was the expected phenotype of an STR-associated disease, leading to the assumption that this variant was benign and unrelated to corneal dystrophy [74]. Most patients with FECD3 show expanded alleles (68–76%), but penetrance is incomplete, as expanded alleles are also found in 3–6% of unaffected individuals. As such, the 4.21% PG percentage for *TCF4* in the gnomAD cohort is plausible in the context of known biology.

Conversely to the late-onset FECD3, Duchenne muscular dystrophy (DMD) is a severe, progressive disease with motor symptoms typically by age 2–3. Most patients are wheelchair dependent after the first decade of life. One published report links an STR expansion to DMD, and the *DMD* locus is thus included in catalogs of TR diseases such as gnomAD. Given the early onset of DMD, it would be an unexpected causative variant in an adult patient. Similarly, we expect no *DMD* PGs in the gnomAD cohort, although females might be carriers of expanded alleles (≥ 59 repeats). The expected absence of *DMD* is furthered by its relatively rare prevalence: < 1 per 10,000 in males and < 1 per million in females [77].

Instead, the *DMD* locus in males has our study's highest PG percentage (4.705%, ~ 1 per 20 males). A PG is identified in 0.089% of gnomAD females (~ 1 per 1,000), and 8.198% of females are carriers of an expanded allele. Furthermore, the presence of expanded alleles across cohort sex is replicated in the long-read HPRC data. Two males (2/52, 3.85%) and two females (2/48, 4.17%) had PGs in this dataset, and six females were carriers (12.5%). These data contrast dramatically with the disease prevalence of < 1 per 10,000 in males and < 1 per million in females [77].

### Evaluating the locus

In addition to evaluating a specific variant, we can also leverage STRchive to evaluate whether a locus is truly disease relevant. We report the independent observations associated with each locus in addition to the number of PMIDs to show the general level of evidence for each disease (Fig. 2). The well-studied *HTT* locus linked to Huntington’s disease has notably more publications than any other locus, with thousands of cases supporting its characterization. In contrast, more recently discovered loci such as *STARD7* or loci with tenuous evidence for pathogenicity, such as the *DMD* STR locus, have far fewer associated PMIDs and independent observations. By assessing a TR variant alongside its locus, diagnostic teams can prioritize and deprioritize putative variants as appropriate.

### Evidence of TR clinical relevance varies substantially by locus

Presented with a disease of early, severe symptoms juxtaposed with an insupportably high PG percentage (4.705/0.089% in gnomAD and 3.85/4.17% in the long-read HPRC data in males and females, respectively), it is worth evaluating the validity of a causal role for the STR expansion at the *DMD* locus [17]. The proposed PG percentages in the short- and long-read data become even more inconsistent with population prevalence estimates when considering the contribution of other variant classes within the *DMD* gene as a whole to the overall disease burden. The majority (~ 2/3rds) of causative variants underlying DMD are deletions of one or more exons, with the second greatest pathogenic contribution from partial duplications (~ 10%), and then, other variant classes such as missense variants [78]. We would expect STR expansions causing DMD to be far rarer than the general prevalence of DMD, given the commonness of other variant types. These STR expansions being far more common than the prevalence of all pathogenic DMD variants combined indicates that expansions at the *DMD* locus are unlikely to be pathogenic.

As such, it is necessary to interrogate the *DMD* TR locus and its proposed disease relevance. The primary non-experimental method to do so is literature review, which is facilitated by STRchive’s automated literature retrieval. *DMD* a highly repetitive gene, and cataloged literature discuss TRs as markers in linkage and carrier analysis. Nevertheless, only the single case report identifies a “dynamic” expansion of 59–82 repeats through three generations of a pedigree segregating DMD [79]. The impact of this variant on the disease phenotype is speculated without mechanistic validation. No additional studies support the contribution of STR expansion on a DMD phenotype, even when assessing over a thousand individuals with hundreds of heterogeneous variants [80, 81]. In fact, a study genotyping long-read data from 878 individuals within the 1000 Genomes Project found 28 males (6.53%, 28/429) and four females (0.90%, 4/446) with theoretically pathogenic genotypes, as well as 21 female carriers (4.71%) (Additional File 2: Supplementary Methods) [82]. Furthermore, when stratifying *DMD* PG by ancestry within the gnomAD data, there is substantial variation; this may suggest that “expanded” alleles are more suggestive of inherited variants rather than pathogenicity (Additional File 1: Fig. S7). We thus present an additional cohort analysis to refute *DMD* as an STR disease loci, the largest such study to date. Our evidence for refuting the *DMD* STR locus’ role in disease underscores the need for a responsive and dynamic database of STR disease loci that can integrate up-to-date information to ensure reliability.

Although the singular report of *DMD*’s TR association is disparate from established disease loci such as *HTT* and *C9orf72* (Fig. 2), there are additional loci with limited literature such as *ZIC2*, *AFF3*, and *ZNF713*. Furthermore, novel loci will continue to be discovered and require interrogation despite an absence of comparative data. Innovative strategies may be necessary to evaluate pathogenicity, such as assessing genomic region (e.g., coding versus non-coding, overlap with genetic elements) and gene association with disease for nearby non-TR variants. Pathogenicity may also be predicted by tools such as RExPRT [83]. Ultimately, clinical teams must exercise their best judgment and leverage available literature and databases when prioritizing likely TR variants. STRchive consolidates these resources to expedite locus and variant analysis and will mature alongside the TR field.

## Conclusions

STRchive is a comprehensive yet digestible resource of TR Mendelian disease loci. Given its infrastructure within GitHub, STRchive is poised for ongoing revision. Our database can quickly and easily incorporate vetted community contributions outside of regular maintenance to avoid the frustrations of “abandonware” [16]. Even so, STRchive is a manually curated database of a rapidly evolving field. Although information is cited and cross-referenced across resources and by multiple experts, these data are snapshots of TR biology and clinical understandings, subject to clarification and evolution as research progresses. We are not exempt from the abounding complexities of TR genetic variation; users should check underlying evidence linked in STRchive and present in our collected literature. Concerning the aggregate cohort of gnomAD, we lack granular data such as age and PCR status for the majority of samples that could otherwise discretize our analysis of presumably non-penetrant expanded alleles. We also lack genotype data from some STRchive loci not present in gnomAD, precluding PG analysis at these loci.

### Capturing complexity for diagnostic empowerment

Almost half of STRchive 2.0.0 loci are exonic trinucleotide repeats, which may reflect a tendency in locus identification toward coding regions with comparable mechanisms to known diseases [30, 84]. However, as molecular and computational techniques develop, disease loci of greater unorthodoxy are likely to be discovered. In fact, the TR disease loci that have evaded discovery so far are likely to present with increased biological complexity, such as having multiple motifs, interruptions, allele size far exceeding the read length, occurrence at novel repeat loci, and complex locus structures [58]. This shift is exemplified by recent discoveries such as the *RFC1* STR expansions causing CANVAS, which have multiple pathogenic motifs [58]. RExPRT identified ~ 30,000 TR loci in the genome as candidates for pathogenicity [83], suggesting that there are numerous additional disease loci and associated attributes to discover and integrate into STRchive.

TR pathogenic variants are proposed to explain some of the missing heritability in rare disease [20, 85], in part because STRs have mutation rates that are orders of magnitude higher than any other variant class [4, 86]. Additionally, up to 70% of individuals with neurological conditions remain genetically undiagnosed [10], and TR disease loci are frequent causes of neuromuscular and neurodegenerative diseases. By improving the detection and interpretation of TR variants, clinical teams have the potential to provide informative diagnoses [11]. STRchive offers expansive catalogs for multiple reference alignments designed to maximize variant capture. As new pathogenic loci are discovered (and documented within STRchive), their inclusion in rare disease workflows may lead to narrowed diagnostic gaps, clinically actionable outcomes, and shortened diagnostic odysseys [17]. We anticipate that centralizing information within STRchive will improve the standardization of pathogenic thresholds across clinical laboratories, which, in turn, facilitates more efficient diagnostic processes.

Furthermore, we offer a diagnostic blueprint to guide clinical teams through evaluating allele(s) and prioritization of genotypes for further consideration (Table 1). Validation methods are frequently used to confirm TR expansions [15, 87, 88], and intentional evaluation as outlined can prioritize variants warranting resource-intensive follow-up. We provide evidence to endorse TR inclusion in instances where they are often diagnostically excluded, such as in pediatric workflows due to concerns over secondary findings. Specifically, studies often presume that TR diseases are high penetrance conditions, with adult-onset and limited actionability. This has likely led to systematic underdiagnosis of TR diseases in children and young adults. However, TRs are a common and potentially disproportionate cause of phenotypes frequently found in pediatric disease, such as ataxia [89, 90]. Our data also indicate that the majority of TR diseases can have pediatric onset (Fig. 3). With regard to actionability, some TR conditions have treatments in the early stages of development that may benefit patients, and diagnosis may be useful for family planning [91–93]. Lastly, ending the diagnostic odyssey and incorrect diagnoses is often of intrinsic value to patients. As such, testing of relevant TR loci should be incorporated where clinical symptoms warrant further interrogation.

### Inferences made possible through cohort data

We found PG percentages to be broadly higher than disease prevalences estimated for the general populace (Additional File 3: Table S1). There are multiple possibilities for this variation, both biological and technical. The documented pathogenic threshold may be inaccurately defined, or disease penetrance may be lower when alleles are only slightly above the threshold. Prevalence might vary by ancestry and gnomAD subpopulations allelic distributions could differ from general estimates; for example, the STR locus within *DMD* [94] (Additional File 1: Fig. S7). Modifier alleles or changes in sequence composition may lead to reduced penetrance or delayed disease onset [20]. Finally, despite efforts to call all genotypes accurately, certain loci may be subject to increased error rates that require long-read sequencing or higher read coverage to resolve.

However, the concordance between PG estimates across the TOPMed, 100kGP, and gnomAD cohorts suggests these allelic frequencies are generally accurate. This raises several considerations. *Firstly,* it exemplifies how pathogenicity thresholds for TR disease loci remain subject to ongoing investigation and debate while profoundly impacting results [11]. Additional large-scale studies of diverse ancestries are necessary to fully characterize benign, intermediate, and pathogenic allelic ranges. *Secondly,* our work and that of Ibañez et al. suggest that allele size alone may be insufficient to diagnose TR disease, as even expansions that are rare by allelic frequency are found in healthy controls [83]. Population-scale characterization of expanded alleles at loci believed to be completely penetrant has revealed PGs in unaffected individuals, and again, further characterization is necessary [16]. *Lastly,* the *FXN* result hints at the population-specific components of TR disease. While most TR loci expansions are observed across ancestries [28], TRs are observed to vary in frequency and length distributions across ancestral groups [17]. Inconsistencies in pathogenic thresholds may partly be due to population-specific allele distributions and disease penetrance [20]. While most population-scale studies to date have either focused on European ancestry cohorts or been limited by sequencing depth [29], STRchive is positioned to incorporate updates as the above considerations are resolved.

### The future of TR disease loci

The pace of TR discovery and characterization is likely to continue accelerating as sequencing and bioinformatic techniques further evolve [74]. There are several immediate opportunities for innovation. TRs are found across the genome in low-complexity regions such as centromeres and telomeres, which are difficult to interrogate with short-read sequencing [66]. Additionally, while long-read sequencing resolves the issue of expansions exceeding read lengths, it introduces new problems such as stutter, and remains prohibitively expensive [16, 88]. In parallel with the evolution of molecular and computational techniques, studies evaluating control and disease cases to characterize human variation will elucidate known and novel loci alike. There may be opportunities to directly compare pathogenic and non-pathogenic cases in large population databases of diverse ancestries, such as All of Us [95, 96]. Additional features of repeat sequences, such as methylation and mosaicism, may be assayed as made possible by new technologies [15]. Although most studies to date have been largely observational, it is conceivable that therapeutics development will follow the increased characterization of disease loci, particularly as pathogenic mechanisms become better understood [8]. As a comprehensive and dynamic resource, STRchive is positioned to support current and future initiatives addressing TR disease, from empowering resolution to long-standing diagnostic odysseys to guiding projects currently in their infancy.

## Supplementary Information

Supplementary Material 1. Fig. S1. Process figure showing the construction (steps 3–5) and maintenance (1–5) of STRchive as a resource. Fig. S2. Most coding TRs result in polyalanine and polyglutamine tracts. Fig. S3. RFC1 has the highest motif diversity of the gnomAD dataset, with motifs of all classifications. Fig. S4. Six TR disease loci show motif heterogeneity across gnomAD and HPRC cohorts. Fig. S5. Nine loci show motif heterogeneity within HPRC cohort, six with motifs previously not documented. Fig. S6. Comparisons between gnomAD, TOPMed, and 100kGP show general concordance in PG percentage (inset range 0–0.27). Fig. S7. DMD locus shows variation in PG percentage by ancestry.

Supplementary Material 2.

Supplementary Material 3. Table S1. PGs are calculated at gnomAD TR disease loci and compared to disease prevalence, where known. Table S2. Within gnomAD, PGs are found within 14 autosomal dominant loci and two X-linked recessive loci. Table S3. The exact PG percentages within 100kpG and TOPMed show proximity to the gnomAD confidence intervals.

## Data Availability

STRchive is licensed under a Creative Commons Attribution 4.0 International License. STRchive is available at http://strchive.org/, with comprehensive data, metadata, and processing scripts available at https://github.com/dashnowlab/STRchive. All scripts for manuscript data analysis and figure generation are available at https://github.com/dashnowlab/STRchive_manuscript; publicly available data used for analyses is also hosted on this GitHub. gnomAD tandem repeat data, including allele frequency distributions, per-sample genotypes, and other sample metadata, can be explored online at https://gnomad.broadinstitute.org/short-tandem-repeats?dataset=gnomad_r3 and is also available for download on the gnomAD website under “v3 Downloads > Short Tandem Repeats”: gnomad.broadinstitute.org/downloads#v3-short-tandem-repeats [36]. The long-read data from the Human Pangenome Reference Consortium is available from SRA project PRJNA701308 or https://humanpangenome.org/data/ [57].
