# Supplementary material for: STRchive: a dynamic resource detailing population-level and locus-specific insights at tandem repeat disease loci
Source: Genome Med. 2025 Mar 26;17:29. doi: 10.1186/s13073-025-01454-4 (PMC11938676; doi:10.1186/s13073-025-01454-4)
Supplement: Supplementary file 1 — Supplementary Material 1. Fig. S1. Process figure showing the construction (steps 3–5) and maintenance (1–5) of STRchive as a resource. Fig. S2. Most coding TRs result in polyalanine and polyglutamine tracts. Fig. S3. RFC1 has the highest motif diversity of the gnomAD dataset, with motifs of all classifications. Fig. S4. Six TR disease loci show motif heterogeneity across gnomAD and HPRC cohorts. Fig. S5. Nine loci show motif heterogeneity within HPRC cohort, six with motifs previously not documented. Fig. S6. Comparisons between gnomAD, TOPMed, and 100kGP show general concordance in PG percentage (inset range 0–0.27). Fig. S7. DMD locus shows variation in PG percentage by ancestry. [file 13073_2025_1454_MOESM1_ESM.pdf]

Supplementary Figures

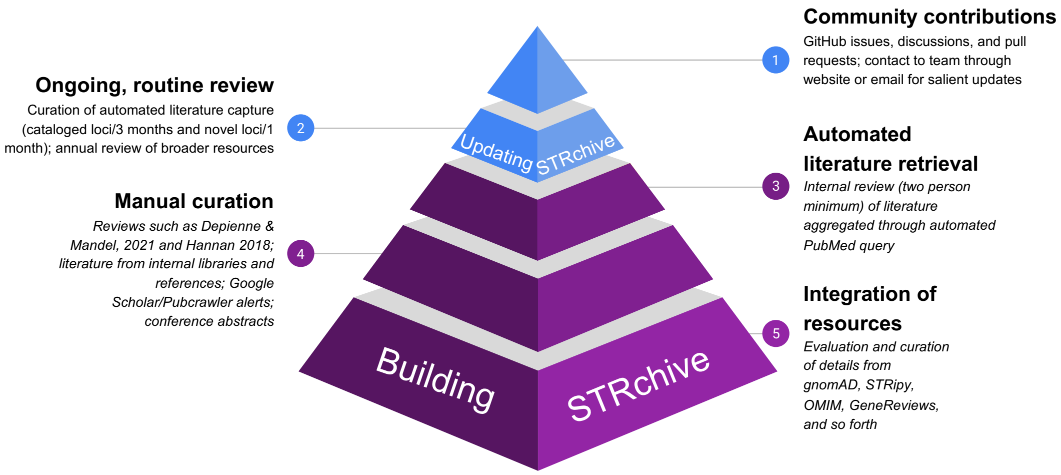

Fig. S1: Process figure showing the construction (steps 3-5) and maintenance (1-5) of STRchive as a resource.

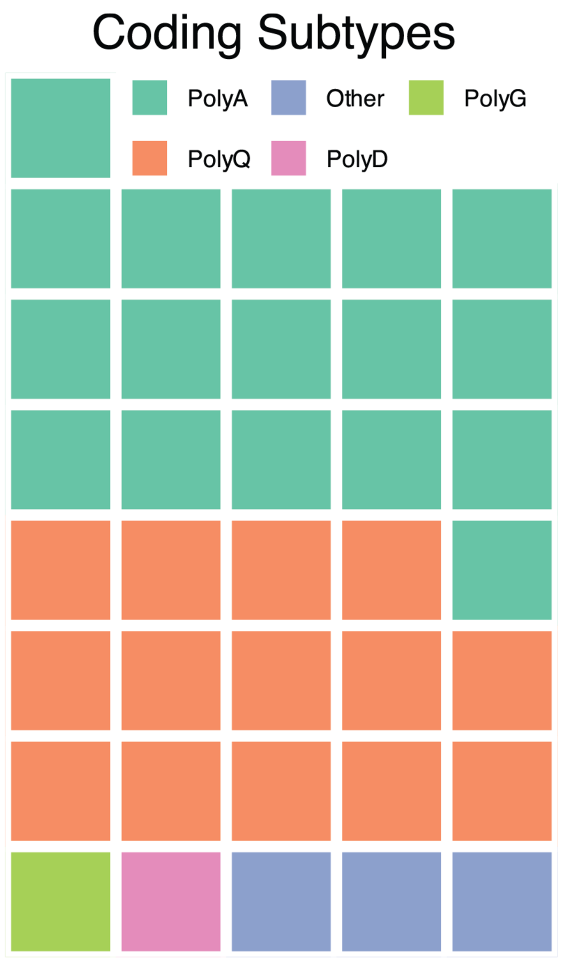

Fig. S2: Most coding TRs result in polyalanine and polyglutamine tracts.
A waffle plot of the coding types' specific coding consequence, where each block represents a locus in STRchive. Script for generation (with further details of the other categories) is available in Figure 1 script.
PolyA: polyalanine; PolyG: polyglycine; PolyQ: polyglutamine; Other: amino acid patterns that do not fall under the previously listed categories.
MUC1 is not included as it is a homopolymer repeat of variable location within a VNTR cluster.

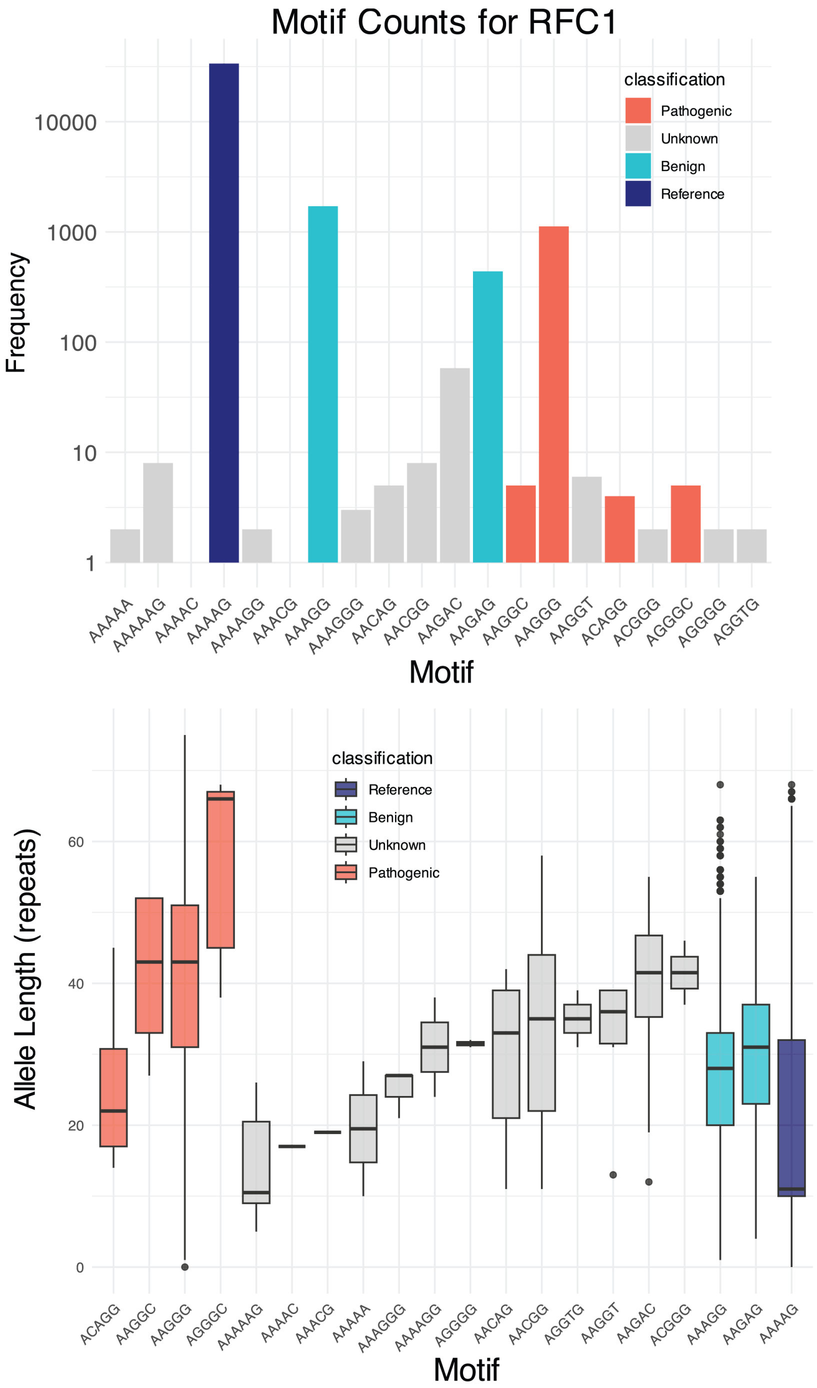

Fig. S3: RFC1 has the highest motif diversity of the gnomAD dataset, with motifs of all classifications. RFC1 motif counts from the gnomAD data for all motifs colored by clinical classification, which show a relatively high proportion of pathogenic motifs as well as a collectively high proportion of unknown motifs. **A.** Motif frequency for the gnomAD dataset. **B.** Motifs plotted by allele length and ordered by median allele size. Plot scripts are available in script for Figure 5 generation.

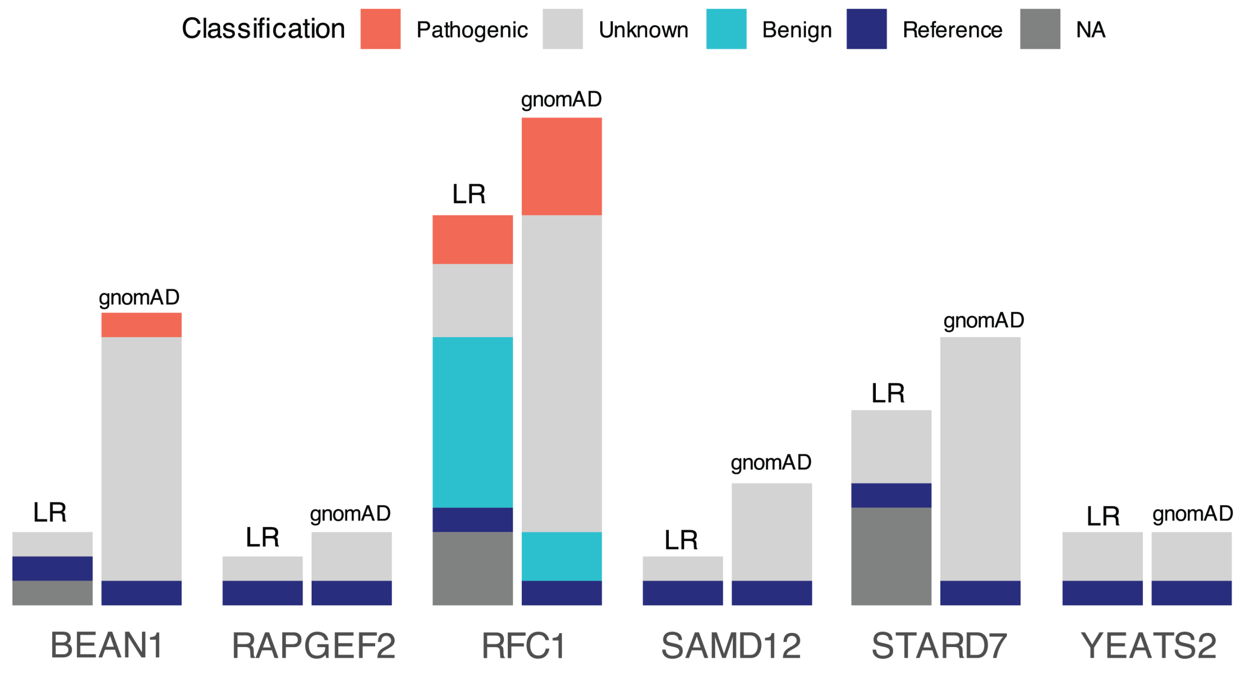

Fig. S4: Six TR disease loci show motif heterogeneity across gnomAD and HPRC cohorts. Unique motifs taken from the HPRC long-read (LR) data compared to the gnomAD data at overlapping STRchive loci. NA indicates “Not Available” in STRchive; other “Unknown” motifs have been documented in the literature before despite not being associated with a phenotype. Script is available in script for Figure 5 generation.

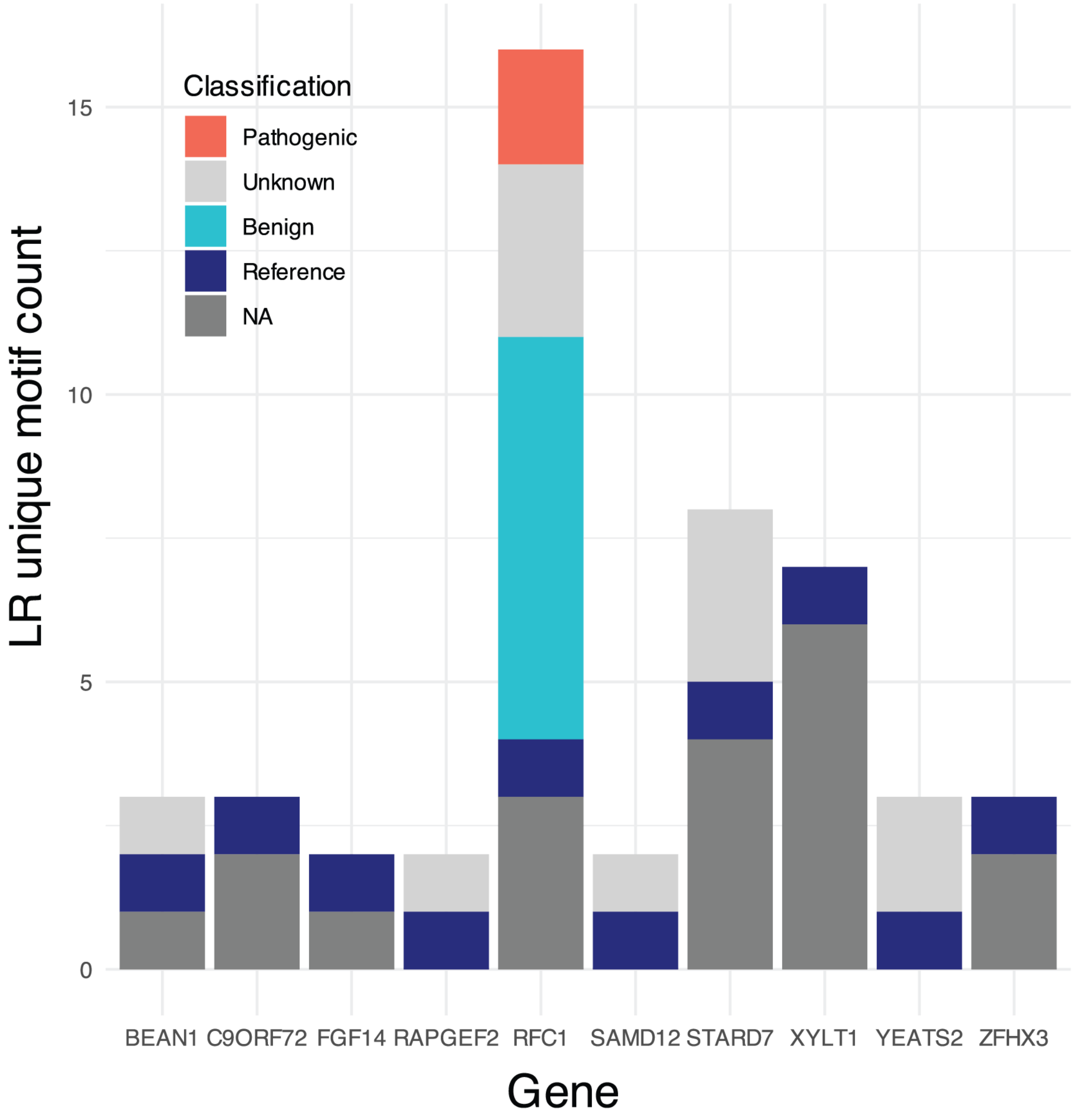

Fig. S5: Nine loci show motif heterogeneity within HPRC cohort, six with motifs previously not documented. Unique motifs taken from the HPRC long-read data, classified by STRchive, for all loci with more than one unique motif. NA indicates “Not Available” in STRchive; other “Unknown” motifs have been documented in the literature before despite not being associated with a phenotype. Script is available in script for Figure 5 generation.

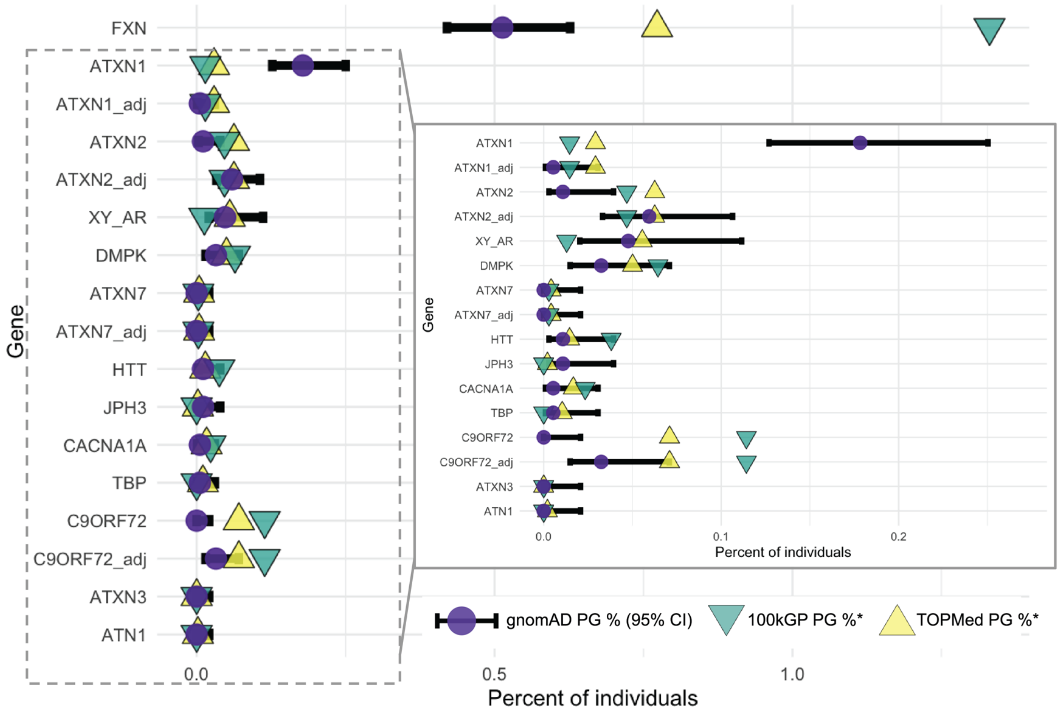

Fig. S6: Comparisons between gnomAD, TOPMed, and 100kGP show general concordance in PG percentage (inset range 0-0.27). An asterisk for PG % is used because FXN is an autosomal recessive locus, and so carrier percentage is used across cohorts rather than pathogenic genotypes. Matching the pathogenic thresholds in gnomAD to what was used by Ibañez et al. for four non-equivalent loci (*ATXN1, ATXN7, ATXN2, C9ORF72*) is indicated by *_adj. This adjustment altered the PG confidence interval to span the TOPMed/100kGP PG percentages for the first three loci while adjusting the pathogenic threshold for *C9ORF72* brought the TOPMed within 0.00018798% of the upper limit of the 95% confidence interval. The TOPMed estimate for *JPH3* was within 0.000879498% of the lower limit of the 95% confidence interval. Curiously, the gnomAD *FXN* PG percentage is comparable to disease prevalence in the literature despite being 37 to 67-fold lower than TOPMed/100kGP estimates. The *FXN* analysis within TOPMed and 100kGP shows ancestry-based PG variation, which aligns with variation in prevalence estimates across different populations reported previously for *FXN*. Consequently, the extreme variation in *FXN* PG percentages across studies may be due to cohort-specific ancestries.

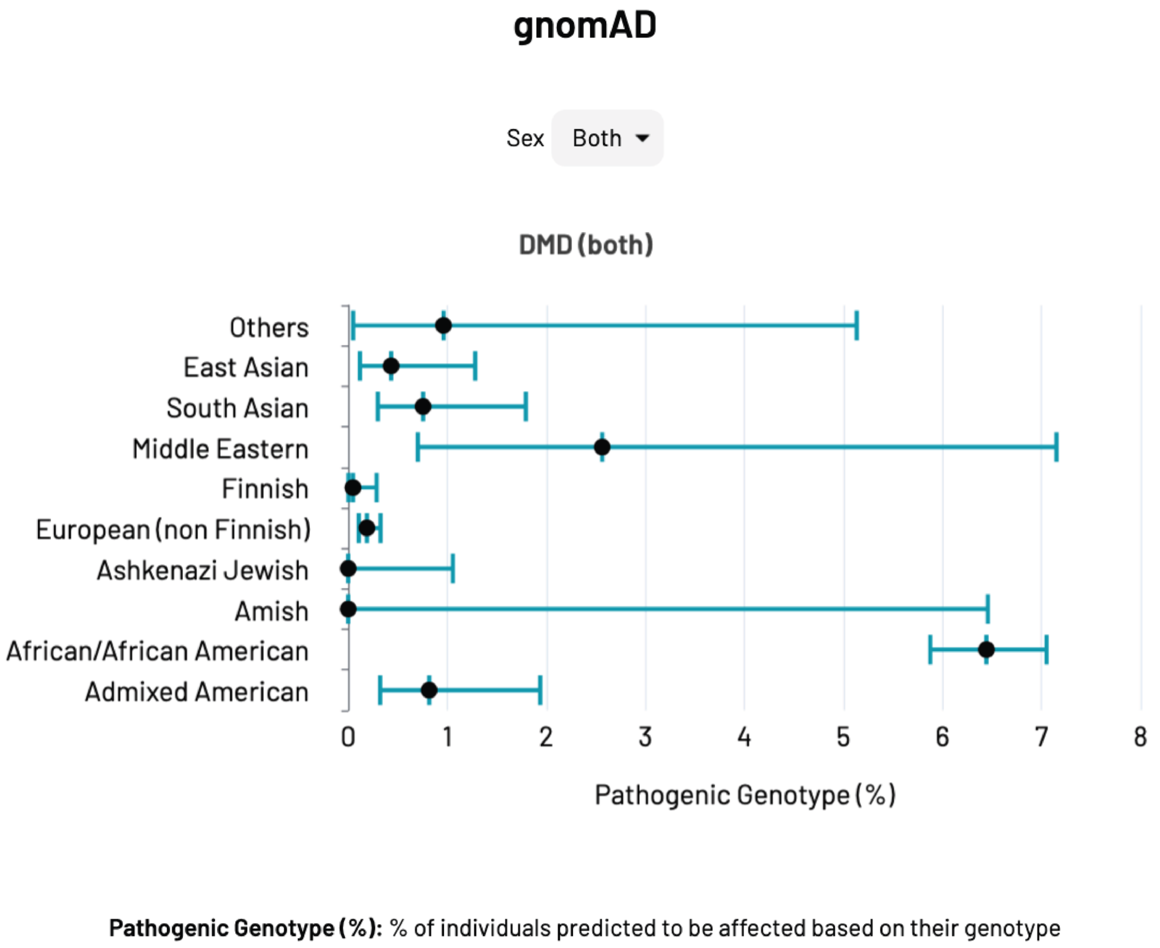

Fig. S7: *DMD* locus shows variation in PG percentage by ancestry. Figure is taken from STRchive.org *DMD* locus page with both sexes represented. The black circle indicates the pathogenic genotype percentage, and the green range shows the 95% confidence interval.
