# Supplementary material for: STRchive: a dynamic resource detailing population-level and locus-specific insights at tandem repeat disease loci
Source: Genome Med. 2025 Mar 26;17:29. doi: 10.1186/s13073-025-01454-4 (PMC11938676; doi:10.1186/s13073-025-01454-4)
Supplement: Supplementary file 3 — Supplementary Material 3. Table S1. PGs are calculated at gnomAD TR disease loci and compared to disease prevalence, where known. Table S2. Within gnomAD, PGs are found within 14 autosomal dominant loci and two X-linked recessive loci. Table S3. The exact PG percentages within 100kpG and TOPMed show proximity to the gnomAD confidence intervals. [file 13073_2025_1454_MOESM3_ESM.pdf]

Table S1: PGs are calculated at gnomAD TR disease loci and compared to disease prevalence, where known. *All gnomAD loci where PGs were calculated, with PG percentage, carrier percentage, prevalence, and 95% binomial confidence interval shown. All numbers are rounded to four decimal places of percentage value. Data generated by CalculatingPGsandConfidenceIntervals.R.*

Gene (AD)	gnomAD PG % (95% CI)	Disease Prevalence %
TCF4	4.2090 (3.9291–4.5079)	4.5000
ATXN8OS	0.5133 (0.4201–0.6270)	0.0005
PRNP	0.3188 (0.2472–0.4109)	*NA*
ATXN1	0.1783 (0.1270–0.2503)	0.0015
DMPK	0.0324 (0.0149–0.0707)	0.0093
GIPC1	0.0162 (0.0055–0.0477)	*NA*
ATXN2	0.0108 (0.0029–0.0394)	0.0015
HTT	0.0108 (0.0029–0.0394)	0.0100
CNBP	0.0108 (0.0029–0.0394)	0.0023
JPH3	0.0108 (0.0029–0.0394)	*NA*
CACNA1A	0.0054 (0.0001–0.0306)	0.0027
COMP	0.0054 (0.0001–0.0306)	*NA*
ATN1	0 (0–0.0208)	0.0005
ATXN10	0 (0–0.0208)	*NA*
ATXN3	0 (0–0.0208)	0.0021
ATXN7	0 (0–0.0208)	0.0003
BEAN1	0 (0–0.0208)	*NA*
C9ORF72	0 (0–0.0208)	*NA*
DAB1	0 (0–0.0208)	*NA*
DIP2B	0 (0–0.0208)	*NA*
LRP12	0 (0–0.0208)	*NA*
MARCHF6	0 (0–0.0208)	*NA*
NOP56	0 (0–0.0208)	*NA*
NUTM2B-AS1	0 (0–0.0208)	*NA*
PPP2R2B	0 (0–0.0208)	*NA*
RAPGEF2	0 (0–0.0208)	*NA*
RILPL1	0 (0–0.0208)	*NA*
SAMD12	0 (0–0.0208)	*NA*
STARD7	0 (0–0.0208)	*NA*

TNRC6A	0 (0–0.0208)	*NA*
YEATS2	0 (0–0.0208)	*NA*

Gene (AR)	gnomAD Carrier % (95% CI)	gnomAD PG % (95% CI)	Disease Prevalence %
FXN	0.5133 (0.4201–0.6270)	0 (0–0.0208)	0.0020
VWA1	0.1189 (0.0785–0.1799)	0 (0–0.0208)	*NA*
PRDM12	0.0432 (0.0219–0.0853)	0 (0–0.0208)	*NA*
XYLT1	0.0108 (0.0030–0.0394)	0 (0–0.0208)	*NA*
CSTB	0.0054 (0.0010–0.0306)	0 (0–0.0208)	*NA*
GLS	0 (0–0.0208)	0 (0–0.0208)	*NA*
EIF4A3	0 (0–0.0208)	0 (0–0.0208)	*NA*
RFC1	0 (0–0.0208)	0 (0–0.0208)	*NA*

Gene (XR)	Sex	gnomAD Carrier % (95% CI)	gnomAD PG % (95% CI)	Disease Prevalence %
DMD	XY		4.7048 (4.3161–5.1266)	0.0048
DMD	XX	8.1984 (7.6137–8.8237)	0.0886 (0.0429–0.1827)	*NA*
AR	XY		0.0477 (0.0204–0.1115)	0.0033
AR	XX	0.0633 (0.0270–0.1480)	0 (0–0.0486)	*NA*

Gene (XD)	gnomAD PG % (95% CI)	Disease Prevalence %
FMR1	0 (0–0.0208)	0.014

Table S2: Within gnomAD, PGs are found within 14 autosomal dominant loci and two X-linked recessive loci. *gnomAD loci where at least one PG was found, with PG percentage and 95% binomial confidence interval shown in contrast with prevalence. All numbers are rounded to four decimal places of percentage value. Literature prevalence is bolded where it is within the confidence interval of the gnomAD pathogenic genotype (PG) percentage. For all other loci (including all AR and XD loci), prevalence was either unavailable or in the range of* $10^{-(4\text{-}6)}$*. Data generated by CalculatingPGsandConfidenceIntervals.R.*

Gene (AD)	gnomAD PG % (95% CI)	Disease Prevalence %
TCF4	4.2090 (3.9291–4.5079)	4.5000
ATXN8OS	0.5133 (0.4201–0.627)	0.0005
PRNP	0.3188 (0.2472–0.4109)	NA
ATXN1	0.1783 (0.1270–0.2503)	0.0015
PABPN1	0.0756 (0.0451–0.1269)	0.0010
DMPK	0.0324 (0.0149–0.0707)	0.0093
GIPC1	0.0162 (0.0055–0.0477)	NA
ATXN2	0.0108 (0.0029–0.0394)	0.0015
HTT	0.0108 (0.0029–0.0394)	0.0100
CNBP	0.0108 (0.0029–0.0394)	0.0023
HOXD13	0.0108 (0.0029–0.0394)	NA
JPH3	0.0108 (0.0029–0.0394)	NA
CACNA1A	0.0054 (0.0001–0.0306)	0.0027
COMP	0.0054 (0.0001–0.0306)	NA

Gene (XR)	Sex	gnomAD PG % (95% CI)	Disease Prevalence %
DMD	XY	4.7048 (4.3161–5.1266)	0.0048
DMD	XX	0.0886 (0.0429–0.1827)	*NA*
AR	XY	0.0477 (0.0204–0.1115)	0.0033

Table S3: The exact PG percentages within 100kpG and TOPMed show proximity to the gnomAD confidence intervals. PG % represented the pathogenic genotype percentage for each dataset.

gene	PG % (100kGP)	PG % (TOPMed)	PG % (STRchive)	PG % (STRchive) Lower Confidence Interval	PG % (STRchive) Upper Confidence Interval
FXN	1.330798479	0.772817002	0.51329155	0.420108889	0.627013
C9ORF72	0.114198706	0.070904238	0	0	0.020751
C9ORF72_adj	0.114198706	0.070904238	0.032418414	0.01485847	0.070716
DMPK	0.064387731	0.050039615	0.032418414	0.01485847	0.070716
ATXN2	0.046819219	0.062557344	0.010806722	0.002963643	0.039398
ATXN2_adj	0.046819219	0.062557344	0.05943697	0.033192926	0.106409
HTT	0.038037277	0.014589716	0.010806722	0.002963643	0.039398
CACNA1A	0.023404131	0.016674309	0.005403653	0.000953884	0.030605
ATXN1	0.014626298	0.02918369	0.178301275	0.126993503	0.250286
ATXN1_adj	0.014626298	0.02918369	0.005403069	0.000953781	0.030601
XY_AR	0.012930756	0.055530875	0.047659899	0.020359124	0.111529
ATXN7	0.002924917	0.004168056	0	0	0.020751
ATXN7_adj	0.002924917	0.004168056	0	0	0.020751
JPH3	0	0.002083985	0.010806138	0.002963483	0.039396
ATXN3	0	0	0	0	0.020751
ATN1	0	0.002083985	0	0	0.020754
